# Structural Invariants for the Verification of Systems with Parameterized Architectures

**DOI:** 10.1007/978-3-030-45190-5_13

**Published:** 2020-03-13

**Authors:** Marius Bozga, Javier Esparza, Radu Iosif, Joseph Sifakis, Christoph Welzel

**Affiliations:** 8 Johannes Kepler University, Linz, Austria; 9 University of Birmingham, Birmingham, UK; 10 Univ. Grenoble Alpes, CNRS, Grenoble INP, Verimag, Saint-Martin-d’Hères, France; 11 Technische Universität München, München, Germany

## Abstract

We consider parameterized concurrent systems consisting of a finite but unknown number of components, obtained by replicating a given set of finite state automata.

Marius Bozga[1], Javier Esparza[2], Radu Iosif[1], Joseph Sifakis[1] and Christoph Welzel[2]

TACAS Artifact Evaluation 2020 Accepted

We consider parameterized concurrent systems consisting of a finite but unknown number of components, obtained by replicating a given set of finite state automata. Components communicate by executing atomic interactions whose participants update their states simultaneously. We introduce an interaction logic to specify both the type of interactions (e.g. rendez-vous, broadcast) and the topology of the system (e.g. pipeline, ring). The logic can be easily embedded in monadic second order logic of $\kappa \geq 1$ successors (WS$\kappa$S), and is therefore decidable.

Proving safety properties of such a parameterized system, like deadlock freedom or mutual exclusion, requires to infer an inductive invariant that contains all reachable states of all system instances, and no unsafe state. We present a method to automatically synthesize inductive invariants directly from the formula describing the interactions, without costly fixed point iterations. We experimentally prove that this invariant is strong enough to verify safety properties of a large number of systems, including textbook examples (dining philosophers, synchronization schemes), classical mutual exclusion algorithms, cache-coherence protocols and self-stabilization algorithms, for an arbitrary number of components.

### 1 Introduction

The problem of parameterized verification asks whether a system composed of $n$ replicated processes is safe, for all $n \geq 2$. By safety we mean that every execution of the system stays clear of a set of global error configurations, such as deadlocks or mutual exclusion violations. Even if we assume each process to be finite-state and every interaction to be a synchronization of actions without exchange of data, ranging over large or infinite domains, the problem remains challenging because we ask for a general proof of safety that works for any number of processes.

Parameterized verification is undecidable, even if processes only manipulate data from a bounded domain [6]. Various restrictions of communication and architecture[3] define decidable subproblems [18,31,27,5]. Seminal works consider *rendez-vous* communication, with participants placed in a ring [18,27] or a clique [31] of arbitrary size. Recently, MSO-definable graphs (with bounded tree- and clique-width) and point-to-point rendez-vous communication have been considered [5]. Most approaches to define decidable problems focus on manually proving a *cut-off* bound $c \geq 2$ such that

** Institute of Engineering Univ. Grenoble Alpes
[3] We use the term architecture for the shape of the graph along which the interactions take place.

correctness for at most $c$ processes implies correctness for any number of processes [18,27,26,7,34]. Other methods identify systems with well-structured transition relations [31,1,29]. An exhaustive chart of decidability results for verification of parameterized systems is drawn in [12]. When decidability is not of concern, over-approximation and semi-algorithmic techniques such as *regular model checking* [36,2], SMT-based *bounded model checking* [4,21], *abstraction* [10,14] and *automata learning* [19] can be used to deal with more general classes of systems.

The efficiency of a verification method crucially relies on its ability to synthesize an *inductive safety invariant*, i.e., an infinite set of configurations that contains the initial configurations, is closed under the transition relation, and excludes the error configurations. In general, automatically synthesizing invariants requires computationally expensive fixpoint iterations [22]. In the particular case of parameterized systems, invariants can be either *global*, relating the local states of all processes [23], or *modular*, relating the local states of a few processes whose identity is irrelevant [38,20].

***Our Contributions.*** The novelty of the approach described in this paper is three-fold:

1. The architecture of the system is not fixed a priori, but given as a parameter of the verification problem. In fact, we describe parameterized systems using the Behavior-Interaction-Priorities (BIP) framework [9], in which processes are instances of finite-state *component types*, whose interfaces are sets of *ports*, labeling transitions between local states, and interactions are sets of strongly synchronizing ports, described by formulae of an *interaction logic*. An interaction formula captures the architecture of the interactions (pipeline, ring, clique, tree) and the communication scheme (rendez-vous, broadcast), which are not hardcoded, but rather specified by the designer of the system.
2. We synthesize parameterized invariants directly from the interaction formula of a system, without iterating its transition relation. Such invariants depend only on the structure (and not on the operational semantics) of an infinite family of Petri Nets, one for each instance of the system, and are thus *structural* invariants. Essentially, the invariants we infer use the *traps*[4] of the system, which are sets $W$ of local states with the property that, if a process is initially in a state from $W$, then always some process will be in a state from $W$. Following [11,17], we call them (parameterized) *trap invariants*. Computing trap invariants only requires a simple syntactic transformation of the interaction formula and the result is expressed using $\mathsf{WS}\kappa\mathsf{S}$, the weak monadic second order logic of $\kappa \geq 1$ successor functions. Thus invariant computation is very cheap, and the verification problem (proving the emptiness of the intersection between the invariant and the set of error states) is reduced to the unsatisfiability of a $\mathsf{WS}\kappa\mathsf{S}$ formula with a single quantifier alternation. In practice, this check can be carried out quite efficiently by existing tools, such as Mona [33].
3. We refine the approach by considering so called 1-*invariants*, that can also be derived cheaply from the interaction formula of the system. We show that 1-invariants in conjunction with trap invariants successfully verify additional examples.

**Comparison to related work.** Trap invariants have been very successfully used in the verification of non-parameterized systems [11,28,13]. The technique was lifted to parameterized systems in [17], but the work there is only applicable to clique architec-

[4] Called in this way by analogy with the notion of traps for Petri Nets [39].

tures, in which processes are indistinguishable, and the system can be described by one single Petri Net with an infinite family of initial markings. Here, for the first time, we show that the trap technique can be extended to pipelines, token rings and trees, where the system is defined by an infinite family of Petri Nets, each with a different structure. These systems cannot be analyzed using the techniques of [31,1,29], because they do not yield well-structured transition systems. Contrary to [18,27,26,7,34], our approach does not require a manual cut-off proof. Contrary to regular model checking and automata learning [2,19], it does not require any symbolic state-space exploration. Finally, our approach produces an explanation of why the property holds in terms of the trap invariant and 1-invariants used. Summarizing, our approach provides a comparatively cheap technique for parameterized verification, that succeeds in numerous cases. It is ideal as preprocessing step that can very quickly lead to success with a very clear explanation of why the property holds, and otherwise provides at least a strong invariant that can be used for further analysis.

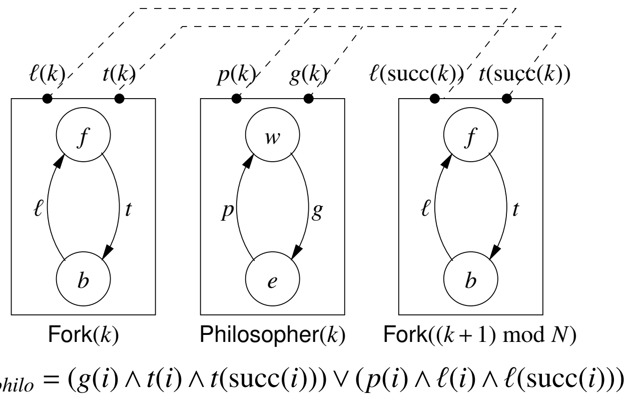

Fig. 1: Parameterized Dining Philosophers

***Running Example.*** Consider the dining philosophers system in Fig. 1, consisting of $n \geq 2$ components of type Fork and Philosopher respectively, placed in a ring of size $2n$. The $k$-th philosopher has a left fork, of index $k$, and a right fork, of index $(k+1) \bmod n$. Each component is an instance of a finite state automaton with states $f$(ree) and $b$(usy) for Fork, respectively $w$(aiting) and $e$(ating) for Philosopher. A fork goes from state $f$ to $b$ via a $t$(ake) transition and from $f$ to $b$ via a $\ell$(eave) transition. A philosopher goes from $w$ to $e$ via a $g$(et) transition and from $e$ to $w$ via a $p$(ut) transition. The $g$ action of the $k$-th philosopher is executed jointly with the $t$ actions of the $k$-th and $[(k+1) \bmod n]$-th forks, in other words, the philosopher takes both its left and right forks simultaneously. Similarly, the $p$ action of the $k$-th philosopher is executed simultaneously with the $\ell$ action of the $k$-th and $[(k+1) \bmod n]$-th forks, i.e. each philosopher leaves both its left and right forks at the same time. We describe these interactions by the *interaction formula*:

$$\Gamma_{philo} = \quad (g(i) \wedge t(i) \wedge t(\mathrm{succ}(i))) \quad \vee \quad (p(i) \wedge \ell(i) \wedge \ell(\mathrm{succ}(i))) \tag{1}$$

where the free variable $i$ refers at some arbitrary component index.

Intuitively, the transitions of the system with $n$ dining philosophers and $n$ forks are given by the *minimal models* of the disjuncts of $\Gamma_{philo}$ with universe $\{0, 1, \ldots, n-1\}$, and succ interpreted as "successor modulo $n$'. In particular, for each $0 \leq k \leq n-1$ the first disjunct has a minimal model that interprets the predicates $g$ and $t$ as the sets $\{k\}$ and $\{k, (k+1) \bmod n\}$. This model describes the interaction in which the $k$-th philosopher takes a $g$-transition (from waiting to eating), while, simultaneously, the $k$-th and $(k+1)$-th forks take $t$-transitions (from free to busy). This is graphically represented by one of the dashed lines in Fig. 1. Observe that the ring topology of the system is implicit in the modulo-$n$ interpretation of the successor function.

Since philosophers can only grab their two forks simultaneously, the system is deadlock-free for any number $n \geq 2$ of philosophers. An automatic proof requires to compute an invariant, and prove that it has an empty intersection with the set of deadlock configurations defined by the WS$\kappa$S formula

$$\begin{aligned} deadlock(X_w, X_e, X_f, X_b) = \forall i \,.\, &[\neg X_w(i) \vee \neg X_f(i) \vee \neg X_f(\mathrm{succ}(i))] \wedge \\ &[\neg X_e(i) \vee \neg X_b(i) \vee \neg X_b(\mathrm{succ}(i))] \end{aligned} \tag{2}$$

where $X_w$, $X_e$, $X_f$, $X_b$ are set variables, the intended meaning of $X_w(i)$ resp. $X_e(i)$ is that the $i$-th philosopher is waiting, resp. eating, and the intended meaning of $X_f(i)$ resp. $X_b(i)$ is that the $i$-th fork is free, resp. busy. Our method automatically computes from $\Gamma_{philo}$ a formula $\textit{trap-invariant}_{\mathcal{S}}$ which formalizes an inductive invariant of the system. Moreover, we express the consistency requirement that every component is in one of its state at all times in a formula $\textit{marking}_{\mathcal{S}}$ and derive the deadlock-freeness for any number of philosophers by the unsatisfiability of the formula

$$deadlock \wedge \textit{trap-invariant}_{\mathcal{S}} \wedge \textit{marking}_{\mathcal{S}}\,.$$

### 2 Parameterized Component-based Systems

A *component type* is a tuple $C = \langle \mathsf{P}, \mathsf{S}, s_0, \Delta \rangle$, where $\mathsf{P} = \{p, q, r, \ldots\}$ is a finite set of *ports*, $\mathsf{S}$ is a finite set of *states*, $s_0 \in \mathsf{S}$ is an initial state and $\Delta \subseteq \mathsf{S} \times \mathsf{P} \times \mathsf{S}$ is a set of *transitions* denoted $s \xrightarrow{p} s'$, for $s, s' \in \mathsf{S}$ and $p \in \mathsf{P}$. We assume there are no two different transitions with the same port.

A *component-based system* $\mathcal{S} = \langle C^1, \ldots, C^N, \Gamma \rangle$ consists of a fixed number $N \geq 1$ of component types $C^k = \langle \mathsf{P}^k, \mathsf{S}^k, {s_0}^k, \Delta^k \rangle$ and an *interaction formula* $\Gamma$. In the dining philosophers there are two component types, Philosopher and Fork, each with two states and two transitions, as shown in Fig. 1. We assume that $\mathsf{P}^i \cap \mathsf{P}^j = \emptyset$ and $\mathsf{S}^i \cap \mathsf{S}^j = \emptyset$, for all $1 \leq i < j \leq N$. We denote the component type of a port $p$ or a state $s$ by $type(p)$ and $type(s)$, respectively. For instance, in Fig. 1 we have $type(p) = type(g) = type(w) = type(e) = $ Philosopher and $type(t) = type(\ell) = type(f) = type(b) = $ Fork.

The interaction formula $\Gamma$ determines the family of systems we can construct out of these components. It does so by specifying, for each possible number of replicated instances (for example, 3 philosophers and 3 forks), which are the possible interactions between them. An interaction consists of a set of transitions that are executed simultaneously. For example, in an interaction philosopher 3 executes a $g$(et) transition simultaneously with $t$(ake) transitions of the forks 2 and 3. Before formalizing this, we introduce the syntax and semantics of Interaction Logic.

**Interaction Logic.** For a constant $\kappa \geq 1$, fixed throughout the paper, the *Interaction Logic* $\mathsf{IL}\kappa$ is built on top of a countably infinite set $\mathsf{Var}$ of variables, the set $\mathsf{Pred} = \bigcup_{k=1}^{N} \mathsf{P}^k$ of monadic predicate symbols ranged over by $\mathsf{pr}$ (i.e. the logic has a predicate symbol for each port), the binary predicate $\leq$, and the *successor* functions $\mathrm{succ}_0$, ..., $\mathrm{succ}_{\kappa-1}$, of arity one. The formulae of $\mathsf{IL}\kappa$ are generated by the syntax

$$t := i \in \mathsf{Var} \mid \mathrm{succ}_0(t) \mid \ldots \mid \mathrm{succ}_{\kappa-1}(t) \qquad \text{terms}$$
$$\phi := t_1 \leq t_2 \mid \mathsf{pr}(t) \mid \phi_1 \wedge \phi_2 \mid \neg\phi_1 \mid \exists i \,.\, \phi_1 \quad \text{formulae}$$

Abbreviations like $t_1 = t_2$, $t_1 < t_2$, $\phi_1 \vee \phi_2$, $\phi_1 \leftrightarrow \phi_2$, and $\forall i \,.\, \phi$ are defined as usual. $\mathsf{IL}\kappa$ is interpreted over finite ranked trees of arity $\kappa$, which we identify with a prefix-closed language of words, also called *nodes*, over the alphabet $\{0,\ldots,\kappa-1\}$. The root of the tree is the empty word $\epsilon$, and the children of $w$ are $w0, w1, \ldots, w(\kappa-1)$. Formally, an *interpretation* or *structure* is a pair $\mathcal{I} = (\mathfrak{U}, \iota)$, where the universe $\mathfrak{U}$ is a tree and $\iota$ assigns a node to each variable and a set of nodes to each predicate in $\mathsf{Pred}$. The predicate $\leq$ and the functions $\mathrm{succ}_0, \ldots, \mathrm{succ}_{k-1}$ have the usual fixed interpretations: If $t$ and $t'$ are interpreted as $w$ and $w'$, then $t_1 \leq t_2$ holds iff $w$ is a prefix of $w'$, and $\mathrm{succ}_i(t)$ is interpreted as the node $wi$, if $wi \in \mathfrak{U}$, and as the root $\epsilon$ otherwise. So, loosely speaking, successor functions wrap around to the root.

When $\kappa = 1$, formulae are interpreted on languages $\{\epsilon, 0, 00, \ldots, 0^{n-1}\}$ for some number $n$. To simplify notation, in this case we assume that they are interpreted over the set $\{0, 1, \ldots, n-1\}$, and $\mathrm{succ}_0$ is the usual successor function on numbers, modulo $n$.

Intuitively, a universe $\mathfrak{U}$ determines an instance of the component-based system, with one instance of each component for each $w \in \mathfrak{U}$. So, for example, for $\kappa = 1$ and $\mathfrak{U} = \{0, 1, 2, \ldots, n-1\}$ in our running example we have philosophers $0, 1, \ldots, n-1$ and forks $0, 1, \ldots, n-1$. Generally, with $\kappa = 1$ we can describe pipeline and token-ring architectures, whereas higher values describe tree-shaped architectures.

**Interaction formulae.** A formula of $\mathsf{IL}\kappa$ is an *interaction formula* if it is the conjunction of the following formula:

$$\forall i \forall j \,.\, \bigwedge_{\substack{p,q \in \mathsf{Pred} \\ type(p)=type(q)}} p(i) \wedge q(j) \rightarrow i \neq j \tag{3}$$

with a finite disjunction of formulae of the form:

$$\mathfrak{C}(i_1, \ldots, i_\ell) \stackrel{\text{def}}{=} \varphi \;\wedge\; \bigwedge_{j=1}^{\ell} p_j(i_j) \;\wedge\; \bigwedge_{j=1}^{m} \forall k \,.\, \psi_j \rightarrow q_j(k) \tag{4}$$

where $\varphi, \psi_1, \ldots, \psi_m$ are conjunctions of atomic formulae of the form $t_1 \leq t_2$ and their negations. Intuitively, formula (3) is a generic axiom that prevents two ports of the same instance of a component type from interacting. The formulae of form (4) are called the *clauses* of the interaction formula.

*Example 1.* Consider a component-based system $\mathcal{S} = \langle C^1, C^2, \Gamma \rangle$, where $C^1$ and $C^2$ have ports $p_1$ and $p_2$, respectively, and $\Gamma$ has one single clause

$$\mathfrak{C}(i, j, k) = \;(i < j \wedge k = \mathrm{succ}(j)) \;\wedge\; (p_1(i) \wedge p_2(j)) \;\wedge\; \forall i. i > k \rightarrow p_1(i)$$

$\Gamma$ states that an interaction consists of: the $i$-th process of type $C^1$ executes transition $p_1$; the $j$-th process of type $C^2$ executes $p_2$; and, for every $i > (j+1) \bmod n$, the $i$-th process of type $C^1$ executes transition $p_1$ as well; all this happens simultaneously in one atomic step. ■

Loosely speaking, (4) states that in an interaction $\ell$ components can simultaneously engage in a multiparty rendez-vous, together with a broadcast to the ports $q_1,\ldots,q_m$ of the components whose indices satisfy the constraints $\psi_1,\ldots,\psi_m$, respectively. An example of peer-to-peer rendez-vous with no broadcast is the dining philosophers system in Fig. 1, whereas examples of broadcast are found among the benchmarks in §5. In the next section we show that, despite this generality, it is possible to construct a trap invariant for any interaction formula in a purely syntactic way.

Observe that the interaction formula does not explicitly specify that every other process remains idle. Formally, as we will see in the next section, the system has an interaction for each *minimal model* of (4), which allows us not to have to specify idleness. Given structures $\mathcal{I}_1 = (\mathfrak{U},\iota_1)$ and $\mathcal{I}_2 = (\mathfrak{U},\iota_2)$ sharing the same universe $\mathfrak{U}$, we say $\mathcal{I}_1 \sqsubseteq \mathcal{I}_2$ if and only if $\iota_1(\mathsf{pr}) \subseteq \iota_2(\mathsf{pr})$ for every $\mathsf{pr} \in \mathsf{Pred}$. Given a formula $\phi$, a structure $\mathcal{I}$ is a *minimal model* of $\phi$ if $\mathcal{I} \models \phi$ and, for all structures $\mathcal{I}'$ such that $\mathcal{I}' \sqsubseteq \mathcal{I}$ and $\mathcal{I}' \neq \mathcal{I}$, we have $\mathcal{I}' \not\models \phi$.

### 2.1 Execution Semantics of Component-based Systems

The semantics of a component-based system $\mathcal{S} = \langle C^1,\ldots,C^N,\Gamma\rangle$ is an infinite family of Petri Nets, one for each universe of $\Gamma$. The reachable markings and actions of the Petri Net characterize the reachable global states and transitions of the system, respectively. To fix notations, we recall several basic definitions.

**Preliminaries: Petri Nets.** A *Petri Net* (PN) is a tuple $\mathsf{N} = \langle S,T,E\rangle$, where $S$ is a set of *places*, $T$ is a set of *transitions*, $S \cap T = \emptyset$, and $E \subseteq (S \times T) \cup (T \times S)$ is a set of *arcs*. The elements of $S \cup T$ are called *nodes*. Given nodes $x,y \in S \cup T$, we write $E(x,y) \stackrel{\mathsf{def}}{=} 1$ if $(x,y) \in E$ and $E(x,y) \stackrel{\mathsf{def}}{=} 0$, otherwise. For a node $x$, let ${}^\bullet x \stackrel{\mathsf{def}}{=} \{y \in S \cup T \mid E(y,x) = 1\}$, $x^\bullet \stackrel{\mathsf{def}}{=} \{y \in S \cup T \mid E(x,y) = 1\}$ and lift these definitions to sets of nodes.

A *marking* of $\mathsf{N}$ is a function $\mathrm{m} : S \to \mathbb{N}$. A transition $t$ is *enabled* in m if and only if $\mathrm{m}(s) > 0$ for each place $s \in {}^\bullet t$. For all markings m, m′ and transitions $t$, we write $\mathrm{m} \xrightarrow{t} \mathrm{m}'$ whenever $t$ is enabled in m and $\mathrm{m}'(s) = \mathrm{m}(s) - E(s,t) + E(t,s)$, for all $s \in S$. Given two markings m and m′, a finite sequence of transitions $\sigma = t_1,\ldots,t_n$ is a *firing sequence*, written $\mathrm{m} \xrightarrow{\sigma} \mathrm{m}'$ if and only if either (i) $n = 0$ and $\mathrm{m} = \mathrm{m}'$, or (ii) $n \geq 1$ and there exist markings $\mathrm{m}_1,\ldots,\mathrm{m}_{n-1}$ such that $\mathrm{m} \xrightarrow{t_1} \mathrm{m}_1 \ldots \mathrm{m}_{n-1} \xrightarrow{t_n} \mathrm{m}'$.

A *marked Petri Net* is a pair $\mathcal{N} = (\mathsf{N},\mathrm{m}_0)$, where $\mathrm{m}_0$ is the *initial marking* of $\mathsf{N}$. A marking m is *reachable* in $\mathcal{N}$ if there exists a firing sequence $\sigma$ such that $\mathrm{m}_0 \xrightarrow{\sigma} \mathrm{m}$. We denote by $\mathcal{R}(\mathcal{N})$ the set of reachable markings of $\mathcal{N}$. A marked PN $\mathcal{N}$ is 1-*safe* if $\mathrm{m}(s) \leq 1$, for each $s \in S$ and $\mathrm{m} \in \mathcal{R}(\mathcal{N})$. All PNs considered in the following will be 1-safe and we shall silently blur the distinction between a marking $\mathrm{m} : S \to \{0,1\}$ and the boolean valuation $\beta_\mathrm{m} : S \to \{\bot,\top\}$ defined as $\beta_\mathrm{m}(s) = \top \iff \mathrm{m}(s) = 1$. A set of markings $\mathcal{M}$ is an *inductive invariant* of $\mathcal{N} = (\mathsf{N},\mathrm{m}_0)$ if and only if $\mathrm{m}_0 \in \mathcal{M}$ and for each $\mathrm{m} \xrightarrow{t} \mathrm{m}'$ such that $\mathrm{m} \in \mathcal{M}$, we have $\mathrm{m}' \in \mathcal{M}$.

**Petri Net Semantics of Component-Based Systems.** We define the semantics of a component-based system as an infinite family of 1-safe Petri Nets. For $k = 1,\ldots,N$ let $C^k = \langle \mathsf{P}^k,\mathsf{S}^k,s_0{}^k,\Delta^k\rangle$ be a component type and, then, let $\mathcal{S} = \langle C^1,\ldots,C^N,\Gamma\rangle$ be a system. Fix a universe $\mathfrak{U}$ of $\Gamma$. We define a marked Petri Net $\mathcal{N}^{\mathfrak{U}}_{\mathcal{S}} \stackrel{\mathsf{def}}{=} (\langle S,T,E\rangle,\mathrm{m}_0)$ as follows:

- $S \stackrel{\text{def}}{=} \left(\bigcup_{k=1}^{N} \mathsf{S}^k\right) \times \mathfrak{U}$. That is, the net has a place $(s,u)$ for each state $s$ of each component type, and for each node $u$.
- For each minimal model $\mathcal{I} = (\mathfrak{U}, \iota)$ of a clause $\mathfrak{C}$ of $\Gamma$, the set $T$ contains a transition $\mathrm{t}_\iota \in T$, and the set $E$ contains edges $((s,u), \mathrm{t}_\iota)$ and $(\mathrm{t}_\iota, (s',u))$ for every $s \xrightarrow{p} s' \in \left(\bigcup_{k=1}^{N} \Delta^k\right)$ such that $u \in \iota(p)$. Nothing else is in $T$ or $E$. Intuitively, $\mathrm{t}_\iota$ "synchronizes" all the transitions $s \xrightarrow{p} s'$ of the different components occurring in the interaction.
- For each $1 \leq k \leq N$, each $s \in \mathsf{S}^k$ and each $u \in \mathfrak{U}$, $\mathrm{m}_0((s,u)) = 1$ if $s = {s_0}^k$ and $\mathrm{m}_0((s,u)) = 0$, otherwise. That is, $\mathrm{m}_0$ contains the places $(s,u)$ such that $s$ is an initial state.

It follows immediately from this definition that $\mathcal{N}_S^{\mathfrak{U}}$ is a 1-safe Petri Net. Indeed, for every $u \in \mathfrak{U}$, for every component-type $C^k$, and for every reachable marking $m$, we have $\sum_{s \in \mathsf{S}^k} m((s,u)) = 1$. This reflects that the instance of $C^k$ at $u$ is always in exactly one of the states of $\mathsf{S}^k$; if $s$ is that state, then $(s,u)$ is the place carrying the token.

*Example 2.* Consider our running example, with $\mathfrak{U} = \{0, 1, \ldots, n-1\}$, i.e., $n$ philosophers and $n$ forks. Since the interaction formula (1) has no constants, its models are pairs $(\mathfrak{U}, \iota)$, where $\iota$ gives the interpretation of the free variable $i$ and the predicates $g$, $t$, etc. The first disjunct of (1) is $[g(i) \wedge t(i) \wedge t(\mathrm{succ}(i))]$. It has a minimal model for each $k \in \mathfrak{U}$, namely the model with $\iota(i) = k$, $\iota(g) = \{k\}$ and $\iota(t) = \{k, (k+1) \bmod n\}$. In the interaction produced by this model, the $k$-th philosopher executes transition $g$(et), the forks with numbers $k$ and $(k+1) \bmod n$ execute transition $t$(ake), and all other philosophers and forks remain idle. The second disjunct yields the interactions in which a philosopher puts down its forks. Fig. 2 shows the Petri Net $\mathcal{N}_S^{\mathfrak{U}}$ for universe $\mathfrak{U} = \{0, 1, 2\}$. For clarity,

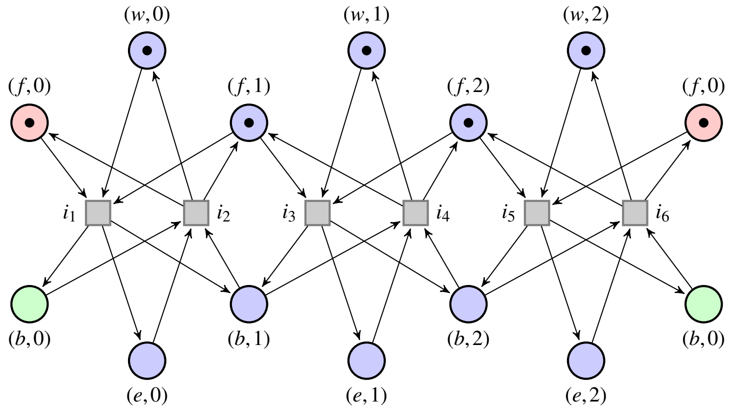

Fig. 2: Petri Net of the dining philosophers for the universe $\mathfrak{U} = \{0, 1, 2\}$. In reality, the two pink and green places are only one place.

the places $(f,0)$ and $(b,0)$ have been duplicated; in reality the two copies are merged. The places of each philosopher are $\{(w,i),(e,i)\}$ for $i = 0, 1, 2$. For example, transition

$i_3$ corresponds to the minimal model $\{(g,1),(t,1),(t,2)\}$, in which philosopher 1 takes forks 1 and 2.

## 3 Trap Invariants

Given a Petri Net $\mathsf{N} = (S,T,E)$, a set of places $W \subseteq S$ is called a *trap* if and only if $W^\bullet \subseteq {}^\bullet W$. A trap $W$ of $\mathsf{N}$ is an *initially marked trap* (IMT) of the marked PN $\mathcal{N} = (\mathsf{N}, \mathrm{m}_0)$ if and only if $\mathrm{m}_0(s) = \top$ for some $s \in W$.

*Example 3.* $\{(f,1),(b,1)\}$ and $\{(f,0),(b,1),(f,2),(e,2)\}$ are two traps of the Petri Net in Figure 2.

An IMT defines an invariant of the Petri Net, because some place in the trap will always be marked, no matter which sequence of transitions is fired. The *trap invariant* of $\mathcal{N}$ is the set of markings that mark each IMT of $\mathcal{N}$. Clearly, since marked traps remain marked, the set of reachable markings is contained in the trap invariant. Hence, to prove that a certain set of markings is unreachable, it is sufficient to prove that the set has empty intersection with the trap invariant. For self-completeness, we briefly discuss the computation of the trap invariant for a given marked Petri Net of fixed size, before explaining how this can be done for the infinite family of marked Petri Nets defining the executions of parameterized systems.

The *trap constraint* of a Petri Net $\mathsf{N} = (S,T,E)$ is the formula:

$$\Theta(\mathsf{N}) \stackrel{\mathsf{def}}{=} \bigwedge_{t\in T} \left(\bigvee_{x \in {}^\bullet t} x\right) \rightarrow \left(\bigvee_{y \in t^\bullet} y\right)$$

where each place $x, y \in S$ is viewed as a propositional variable. It is not hard to show[5] that any boolean valuation $\beta : S \rightarrow \{\bot, \top\}$ that satisfies the trap constraint $\Theta(\mathsf{N})$ defines a trap $W_\beta$ of $\mathsf{N}$ in the obvious sense $W_\beta = \{s \in S \mid \beta(s) = \top\}$. Further, if $\mathrm{m}_0 : S \rightarrow \{0,1\}$ is the initial marking of a 1-safe Petri Net $\mathsf{N}$ and $\mu_0 \stackrel{\mathsf{def}}{=} \bigvee_{\mathrm{m}_0(s)=1} s$ is a propositional formula, then every valuation of $\mu_0 \wedge \Theta(\mathsf{N})$ defines an IMT of $(\mathsf{N}, \mathrm{m}_0)$. Usually, computing invariants requires building a sequence of underapproximants whose limit is the least fixed point of an abstraction of the transition relation of the system [22]. This is not the case of the trap invariant, that can be directly computed from the trap constraint and the initial marking [11,17].

In the rest of the section we construct a parameterized trap constraint that characterizes the traps, not of one single net, as $\Theta(\mathsf{N})$, but of the infinite family of Petri Nets obtained from a component-based system. The parameterized trap constraint is a formula of $\mathsf{WS}\kappa\mathsf{S}$. In Section 3.1 we first explain how to embed our interaction logic into $\mathsf{WS}\kappa\mathsf{S}$, and in Section 3.2 we construct the parameterized trap constraint.

### 3.1 From $\mathsf{IL}\kappa$ to $\mathsf{WS}\kappa\mathsf{S}$

We briefly recall the syntax and semantics of $\mathsf{WS}\kappa\mathsf{S}$, the monadic second order logic $\mathsf{WS}\kappa\mathsf{S}$ of $\kappa$ successors (see e.g. [37]). Let $\mathsf{SVar}$ be a countably infinite set of second-

[5] See e.g. [8] for a proof.

order variables (also called set variables), denoted as $X, Y, \ldots$ in the following. The syntax of WS$\kappa$S is:

$$t := \overline{\epsilon} \mid x \mid \mathrm{succ}_0(t) \mid \ldots \mid \mathrm{succ}_\kappa(t) \quad \text{terms}$$
$$\phi := t_1 = t_2 \mid \mathsf{pr}(t) \mid X(t) \mid \phi_1 \wedge \phi_2 \mid \neg\phi_1 \mid \exists x \,.\, \phi_1 \mid \exists X \,.\, \phi_1 \quad \text{formulae}$$

So WS$\kappa$S extends IL$\kappa$ with the constant symbol $\overline{\epsilon}$, atoms $X(t)$ and monadic second order quantifiers $\exists X \,.\, \phi$. We can consider w.l.o.g. equality atoms $t_1 = t_2$ instead of the inequalities $t_1 \leq t_2$ in IL$\kappa$, because the latter can be defined in WS$\kappa$S as usual:

$$x \leq y \stackrel{\mathsf{def}}{=} \forall X \,.\, closed(X) \wedge X(x) \rightarrow X(y) \qquad closed(X) \stackrel{\mathsf{def}}{=} \forall x \,.\, X(x) \rightarrow \bigwedge_{i=0}^{\kappa-1} X(\mathrm{succ}_i(x))$$

Like IL$\kappa$, the formulae of WS$\kappa$S are interpreted on ordered trees of arity $\kappa$. The models of WS$\kappa$S are structures $(\mathfrak{U}, \iota)$, where $\iota$ assigns the root of the tree to $\overline{\epsilon}$, a node $\iota(x)$ to each variable $x \in \mathsf{Var}$ and a set $\iota(X) \subseteq \mathfrak{U}$ to each set variable $X \in \mathsf{SVar}$. The satisfaction relation $(\mathfrak{U}, \iota) \models_{\mathsf{WS}\kappa\mathsf{S}} \phi$ is defined as for IL$\kappa$, with one difference: in IL$\kappa$, the successor of a leaf of a tree is the root of the tree, while in WS$\kappa$S the successor of leaf is, by convention, the leaf itself [37, Example 2.10.3]. This is the only reason why IL$\kappa$ is not just a fragment of WS$\kappa$S.

We define an embedding of IL$\kappa$ formulae, without occurrences of predicates and set variables, into WS$\kappa$S. W.l.o.g. we consider IL$\kappa$ formulae that have been previously flattened, i.e the successor function occurs only within atomic propositions of the form $\mathrm{succ}_i(x) = y$. This is done by replacing each atomic proposition of the form $\mathrm{succ}_{i_1}(\ldots \mathrm{succ}_{i_n}(x) \ldots) = y$ by the formula $\exists x_1 \ldots \exists x_n \,.\, x_n = \mathrm{succ}_{i_n}(x) \wedge y = \mathrm{succ}_{i_1}(x_1) \wedge \bigwedge_{j=1}^{n-1} x_j = \mathrm{succ}_{i_j}(x_{j+1})$. The translation of an IL$\kappa$ formula $\phi$ into WS$\kappa$S is the formula $Tr(\phi)$, defined recursively on the structure of $\phi$ such that $Tr$ simply preserves first-order connectives and, secondly, yields:

$$Tr(\mathrm{succ}_i(x) = y) \stackrel{\mathsf{def}}{=} (\neg \max(x) \wedge \mathrm{succ}_i(x) = y) \vee (\max(x) \wedge y = \overline{\epsilon}).$$

We show that a formula $\phi$ of IL$\kappa$ and its WS$\kappa$S counterpart $Tr(\phi)$ are equivalent:

**Lemma 1.** *Given an* IL$\kappa$ *formula* $\phi$*, for any structure* $\mathcal{I} = (\mathfrak{U}, \iota)$*, we have* $\mathcal{I} \models_{\mathsf{IL}} \phi \iff \mathcal{I} \models_{\mathsf{WS}\kappa\mathsf{S}} Tr(\phi)$.

#### 3.2 Defining Parameterized Trap Invariants in WS$\kappa$S

Fix a component-based system $\mathcal{S} = \langle C^1, \ldots, C^N, \Gamma \rangle$ and recall that every universe $\mathfrak{U}$ induces a Petri Net $\mathsf{N}_{\mathcal{S}}^{\mathfrak{U}}$ whose set of places is $\bigcup_{k=1}^{N} \mathsf{S}^k \times \mathfrak{U}$. For every state $s \in \bigcup_{i=1}^{N} \mathsf{S}^i$, let $X_s$ be a monadic second-order variable, and let $\overline{X}$ be the tuple of these variables in an arbitrary but fixed order. We define a formula $trap\text{-}pred_{\mathcal{S}}(\overline{X})$, with $\overline{X}$ as set of free variables, that characterizes the traps of the infinitely many Petri Nets $\mathsf{N}_{\mathcal{S}}^{\mathfrak{U}}$ corresponding to $\mathcal{S}$. Formally, $trap\text{-}pred_{\mathcal{S}}(\overline{X})$ has the following property:

For every universe $\mathfrak{U}$ and for every set $P \subseteq \bigcup_{k=1}^{N} \mathsf{S}^k \times \mathfrak{U}$ of places of $\mathsf{N}_{\mathcal{S}}^{\mathfrak{U}}$:
$P$ is a trap of $\mathsf{N}_{\mathcal{S}}^{\mathfrak{U}}$ iff the assignment $X_q \mapsto \{u \in \mathfrak{U} \mid (q, u) \in P\}$ satisfies $trap\text{-}pred_{\mathcal{S}}(\overline{X})$.

Observe that every assignment to $\overline{X}$ encodes a set of places, and vice versa. So, abusing language, we can speak of the set of places $\overline{X}$.

We define auxiliary predicates that capture the intersection of the set of places $\overline{X}$ with the pre (${}^\bullet t$) and postset ($t^\bullet$) of a transition $t$ in $\mathsf{N}_{\mathcal{S}}^{\mathfrak{U}}$. For every clause $\mathfrak{C}$ of $\Gamma$, of the form (4), we define the $\mathsf{WS}\kappa\mathsf{S}$ formulae:

$$\textit{intersects-pre}^{\mathfrak{C}}_{\mathcal{S}}(\overline{X}, x_1, \ldots, x_\ell) = \bigvee_{j=1}^{\ell} X_{\bullet p_j}(x_j) \vee \bigvee_{j=\ell+1}^{\ell+m} \exists x_j \,.\, Tr(\psi_j) \wedge X_{\bullet p_j}(x_j) \text{ and}$$

$$\textit{intersects-post}^{\mathfrak{C}}_{\mathcal{S}}(\overline{X}, x_1, \ldots, x_\ell) = \bigvee_{j=1}^{\ell} X_{p_j\bullet}(x_j) \vee \bigvee_{j=\ell+1}^{\ell+m} \exists x_j \,.\, Tr(\psi_j) \wedge X_{p_j\bullet}(x_j).$$

Now we can define $\textit{trap-pred}_{\mathcal{S}}(\overline{X})$ as the conjunction of the following formulae, one for each clause $\mathfrak{C}$ (in the form described in (4)) of $\Gamma$

$$\begin{aligned}\forall x_1 \ldots \forall x_\ell \,.\, \left[Tr(\varphi) \wedge \textit{intersects-pre}^{\mathfrak{C}}_{\mathcal{S}}(\overline{X}, x_1, \ldots, x_\ell)\right] \\ \rightarrow \textit{intersects-post}^{\mathfrak{C}}_{\mathcal{S}}(\overline{X}, x_1, \ldots, x_\ell).\end{aligned} \tag{5}$$

So, intuitively, $\textit{trap-pred}_{\mathcal{S}}(\overline{X})$ states that for every transition of the Petri Net, if the set $\overline{X}$ of places intersects the preset of the transition, then it also intersects its postset. This is the condition for the set of places to be a trap. Formally, we obtain:

**Lemma 2.** *Given a component-based system $\mathcal{S} = \langle C^1, \ldots, C^N, \Gamma\rangle$ and a structure $\mathcal{I} = (\mathfrak{U}, \iota)$, where $\iota$ is an interpretation of the set variables $\overline{X}$, the set $P = \{\langle s, u\rangle \in \bigcup_{k=1}^{N} \mathsf{S}^k \times \mathfrak{U} \mid u \in \iota(X_s)\}$ is a trap of $\mathsf{N}^{\mathfrak{U}}_{\mathcal{S}}$ if and only if $(\mathfrak{U}, \iota) \models_{\mathsf{WS}\kappa\mathsf{S}} \textit{trap-pred}_{\mathcal{S}}(\overline{X})$.*

**Parameterized Trap Invariants in $\mathsf{WS}\kappa\mathsf{S}$.** Loosely speaking, the intended meaning of $\textit{trap-pred}_{\mathcal{S}}(\overline{X})$ is "the set of places $\overline{X}$ is a trap". Our goal is to construct a formula stating: "the marking $m$ marks all initially marked traps".

Recall that the Petri Nets obtained from component-based systems are always 1-safe, and so a marking is also a set of places. Recall, however, that all reachable markings have the property that they place exactly one token in the set of places modeling the set of states of a component (loosely speaking, the set of places of the $k$-th philosopher is $(w,k)$ and $(e,k)$, and there is always one token in the one or the other). So we define a formula $\textit{marking}_{\mathcal{S}}(\overline{X})$ with intended meaning "the set of places $\overline{X}$ is a legal marking", and another one, $\textit{trap-invariant}_{\mathcal{S}}(\overline{X})$ with intended meaning "the set of places $\overline{X}$ marks every initially marked trap".

In addition to the tuple of set variables $\overline{X}$ defined above, we consider now the "copy" tuple $\overline{X'} \stackrel{\text{def}}{=} \langle X'_s\rangle_{s \in \mathsf{S}^i, 1 \le i \le N}$. Intuitively, $\overline{X}$ and $\overline{X'}$ represent one set of places each. First, we define a (1-safe) marking as a set of places that marks exactly one state of each copy of each component:

$$\textit{marking}_{\mathcal{S}}(\overline{X}) = \forall x \,.\, \bigwedge_{1 \le i \le N} \bigvee_{s \in S^i} \left( X_s(x) \wedge \bigwedge_{s' \in S^i \setminus \{s\}} \neg X_{s'}(x) \right).$$

Second, we give a formula describing the intersection of two sets of places:

$$\textit{intersection}_{\mathcal{S}}(\overline{X}, \overline{X'}) = \exists x \,.\, \bigvee_{s \in \bigcup_{1 \le i \le N} \mathsf{S}^i} (X_s(x) \wedge X'_s(x)).$$

Finally, to actually capture IMTs we need to determine if a trap is initially marked. However, this can be easily described by the formula:

$$\textit{initially-marked}_{\mathcal{S}}(\overline{X}) = \exists x \,.\, \bigvee_{1 \le i \le N} X_{s_0{}^i}(x).$$

So we can define the *trap-invariant* by the $\mathsf{WS}\kappa\mathsf{S}$ formula:

$$\begin{aligned}\textit{trap-invariant}_{\mathcal{S}}(\overline{X}) = \forall \overline{X'} \,.\, \left[\textit{trap-pred}_{\mathcal{S}}(\overline{X'}) \wedge \textit{initially-marked}_{\mathcal{S}}(\overline{X'})\right] \\ \rightarrow \textit{intersection}_{\mathcal{S}}(\overline{X}, \overline{X'}).\end{aligned} \tag{6}$$

Relying on Lemma 2 we are assured that the set represented by $\overline{X}$ intersects all IMTs. Now, let $\varphi(\overline{X})$ be any formula that defines a set of *good* global states of the component-based systems (or, equivalently, a good set of markings of their corresponding Petri nets), with the intuition that, at any moment during execution, the current global state of the component-based system should be good. We can now state the following theorem, that captures the soundness of the verification method based on trap invariants:

**Theorem 1.** *Given a component-based system $\mathcal{S}$ and a* WS$\kappa$S *formula $\varphi(\overline{X})$, if the formula*

$$\exists \overline{X} \;.\; marking_{\mathcal{S}}(\overline{X}) \wedge trap\text{-}invariant_{\mathcal{S}}(\overline{X}) \wedge \neg\varphi(\overline{X}) \quad (7)$$

*is unsatisfiable, then for every universe $\mathfrak{U}$, the property defined by the formula $\varphi(\overline{X})$ holds in every reachable marking of $\mathcal{N}_{\mathcal{S}}^{\mathfrak{U}}$.*

In the light of the above theorem, verifying the correctness of a component-based system with any number of active components boils down to deciding the satisfiability of a WS$\kappa$S formula. The latter problem is known to be decidable, albeit with non-elementary worst-case complexity. A closer look at the verification conditions of the form (7) generated by our method suffices to see that the quantifier alternation is finite, which implies that the time needed to decide the (un)satisfiability of (7) is elementary. Moreover, our experiments show that these checks are very fast (less than 1 second on an average machine) for a non-trivial set of examples.

### 4 Refining Trap Invariants

Since the safety verification problem is undecidable for parameterized systems [6], the verification method based on trap invariants cannot be complete. As an example, consider the alternating dining philosophers system, of which an instance (for $n = 3$) is shown in Fig. 3. The system consists of two philosopher component types, namely $\mathsf{Philosopher}_{rl}$, which takes its right fork before its left fork, and $\mathsf{Philosopher}_{lr}$, taking the left fork before the right one. Each philosopher has two interaction ports for taking the forks, namely $g\ell$ (get left) and $gr$ (get right) and one port for releasing the forks $p$ (put). The ports of the $\mathsf{Philosopher}_{rl}$ component type are overlined, in order to be distinguished. The $\mathsf{Fork}$ component type is the same as in Fig. 1. The interaction formula for this system $\Gamma^{alt}_{philo}$, shown in Fig. 3, implicitly states that only the 0-index philosopher component is of type $\mathsf{Philosopher}_{rl}$, whereas all other philosophers are of type $\mathsf{Philosopher}_{lr}$. Note that the interactions on ports $\overline{g\ell}$, $\overline{gr}$ and $\overline{p}$ are only allowed if $zero(x) \stackrel{\mathsf{def}}{=} \forall y \;.\; x \leq y$ holds, in other words if $x$ is interpreted as the root of the universe (in our case, 0 since $\mathfrak{U} = \{0, \ldots, n-1\}$).

It is well-known that any instance of the parameterized alternating dining philosophers system consisting of at least one $\mathsf{Philosopher}_{rl}$ and one $\mathsf{Philosopher}_{lr}$ is deadlock-free. However, trap invariants are not enough to prove deadlock freedom, as shown by the global state $\{\langle b,0\rangle, \langle h,0\rangle, \langle b,1\rangle, \langle w,1\rangle, \langle f,2\rangle, \langle e,2\rangle\}$, marked with thick red lines in Fig. 3. Note that no interaction is enabled in this state. Moreover, this state intersects with any trap of the marked PN that defines the executions of this particular instance, as proved below. Consequently, the trap invariant contains a deadlock configuration, and the system cannot be proved deadlock-free by this method.

Fig. 3: Alternating Dining Philosophers

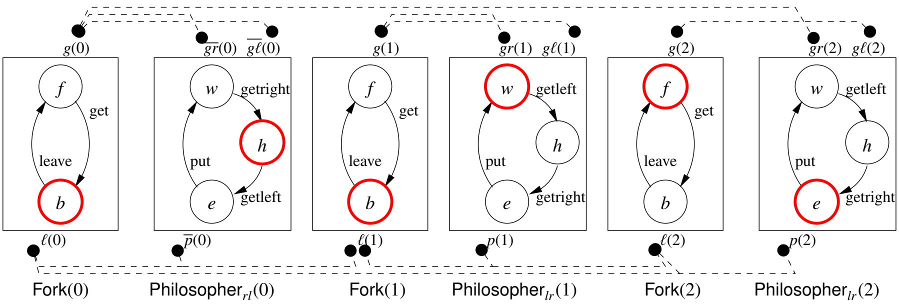

$$\Gamma^{alt}_{philo} = zero(x) \wedge [(\overline{g\ell}(x) \wedge g(x)) \vee (\overline{gr}(x) \wedge g(s(x))) \vee (\overline{p}(x) \wedge \ell(x) \wedge \ell(s(x)))] \vee$$
$$\neg zero(x) \wedge [(g\ell(x) \wedge g(x)) \vee (gr(x) \wedge g(s(x))) \vee (p(x) \wedge \ell(x) \wedge \ell(s(x)))]$$

**Proposition 1.** *Consider an instance of the alternating dining philosophers system in Fig. 3, consisting of components* Fork(0)*,* Philosopher$_{rl}$(0)*,* Fork(1)*,* Philosopher$_{lr}$(1)*,* Fork(2) *and* Philosopher$_{lr}$(2) *placed in a ring, in this order. Then each nonempty trap of this system contains one of the places* $\langle b,0\rangle, \langle h,0\rangle, \langle b,1\rangle, \langle w,1\rangle, \langle f,2\rangle$ *or* $\langle e,2\rangle$.

However, the configuration is unreachable by a real execution of the PN, started in the initial configuration that marks $\langle f,i\rangle$ and $\langle w,i\rangle$, for all $i = 0,1,2$. An intuitive reason is that, in any reachable configuration, each fork is in state $f$(ree) only if none of its neighboring philosophers is in state $e$(ating). In order to prove deadlock freedom, one must learn this and other similar constraints. Next, we present a heuristic method for strengthening the trap invariant that infers such universal constraints.

#### 4.1 One Invariants

As shown by the example above, trap constraints do sometimes fail to prove interesting properties. Hence, it is desirable to refine the overapproximation of viable markings to exclude more spurious counterexamples. In order to do so, we consider a special class of *linear invariants*, called 1*-invariants* in the following. Although linear invariants are not structural and rely on the set of reachable markings of a marked Petri Net, the set of 1-invariants can be sufficiently under-approximated by structural conditions.

**Definition 1.** *Given a marked PN* $\mathcal{N} = ((S,T,E),\mathrm{m}_0)$*, with* $S = \{s_1,\ldots,s_n\}$*, a vector* $\mathbf{a} = (a_1,\ldots,a_n) \in \{0,1\}^n$ *is a* 1-invariant *of* $\mathcal{N}$ *if and only if, for each reachable marking* $\mathrm{m} \in \mathcal{R}(\mathcal{N})$*, we have* $\sum_{i=1}^{n} a_i \cdot \mathrm{m}(s_i) = 1$.

The following lemma relates 1-invariants to some structural properties. However, there are 1-invariants not captured by these conditions. Taking the intersection of this set of 1-invariants defines a weaker invariant, which is sound for our verification purposes.

**Lemma 3.** *Given a marked PN* $\mathcal{N} = ((\mathsf{S},T,E),\mathrm{m}_0)$*, a set of places* $\mathfrak{F} \subseteq \mathsf{S}$ *is a* 1*-invariant if the following hold:*

1. $\sum_{s\in\mathfrak{F}} \mathrm{m}_0(s) = 1$,
2. *either* $\|\mathfrak{F} \cap {}^\bullet t\| = \|\mathfrak{F} \cap t^\bullet\| = k$ *with* $k \in \{0,1\}$ *or* $\|\mathfrak{F} \cap {}^\bullet t\| > 1$ *for every* $t \in T$.

We devote the rest of this section to describe WS$\kappa$S formulae which capture the structural properties necessary to define 1-invariants as laid down by Lemma 3 (2). As demonstrated in Section 3 the pre- and postset of transitions, as well as general sets of places in a PN describing the execution semantics can be defined in WS$\kappa$S. Hence, we present the definitions of the following formulae only in the full version of this article [16] and just give the intuitions here.

As before, we fix two tuples of set variables $\overline{X}$ and $\overline{X'}$, with one variable $X_s$ for each state $s \in \bigcup_{i=1}^{N} \mathsf{S}^i$ and define the following formulae:

- $\mathit{unique\text{-}init}_{\mathcal{S}}(\overline{X})$, which captures that the set of places induced by an interpretation of $\overline{X}$ uniquely intersects the set of all initial states, and
- $\mathit{unique\text{-}intersection}_{\mathcal{S}}(\overline{X},\overline{X'})$, which states that the set of places induced by an interpretation of $\overline{X}$ and $\overline{X'}$ share precisely one place.

Given a transition $t$ of the marked Petri Net $\mathcal{N}_{\mathcal{S}}^{\mathfrak{U}}$ defining the execution semantics of a component-based system $\mathcal{S}$, for a universe $\mathfrak{U}$, we consider the following formulae:

- $\mathit{uniquepre}_{\mathcal{S}}^{\mathfrak{C}}(\overline{X},x_1,\ldots,x_\ell)$, which describes that the set of places encoded by the interpretation of $\overline{X}$ uniquely intersects ${}^\bullet t$, and
- $\mathit{uniquepost}_{\mathcal{S}}^{\mathfrak{C}}(\overline{X},x_1,\ldots,x_\ell)$, which in the same sense captures the unique intersection with $t^\bullet$.

Now we define a predicate $\mathit{1\text{-}pred}_{\mathcal{S}}$ which consists of a conjunction of $\mathit{unique\text{-}init}_{\mathcal{S}}$ and the formulae:

$$\begin{aligned} \forall x_1,\ldots,\forall x_\ell \,.\, (\mathit{Tr}(\varphi) \rightarrow [\neg\, \mathit{intersects\text{-}pre}_{\mathcal{S}}^{\mathfrak{C}} \wedge \neg\, \mathit{intersects\text{-}post}_{\mathcal{S}}^{\mathfrak{C}} \\ \vee\, \mathit{uniquepre}_{\mathcal{S}}^{\mathfrak{C}} \wedge \mathit{uniquepost}_{\mathcal{S}}^{\mathfrak{C}} \\ \vee\, \mathit{intersects\text{-}pre}_{\mathcal{S}}^{\mathfrak{C}} \wedge \neg\, \mathit{uniquepre}_{\mathcal{S}}^{\mathfrak{C}}]) \end{aligned} \tag{8}$$

one for each clause $\mathfrak{C}$ in $\Gamma$. We show the soundness of this definition, by the following:

**Lemma 4.** *Let $\mathcal{S} = \langle C^1,\ldots,C^N,\Gamma\rangle$ be a component-based system and let $\overline{X}$ be a tuple of set variables, one for each state in a component of $\mathcal{S}$. Then, for any structure $(\mathfrak{U},\iota)$ such that $\iota$ interprets the variables in $\overline{X}$, the set $P = \{\langle s,u\rangle \in \bigcup_{i=1}^{N} \mathsf{S}^i \times \mathfrak{U} \mid u \in \iota(X_s)\}$ is a* 1*-invariant of $\mathcal{N}_{\mathcal{S}}^{\mathfrak{U}}$ if $(\mathfrak{U},\iota) \models_{\mathsf{WS\kappa S}} \mathit{1\text{-}pred}_{\mathcal{S}}(\overline{X})$.*

We may now define the 1*-invariant* analogously to the trap-invariant before:

$$\mathit{1\text{-}invariant}_{\mathcal{S}}(\overline{X}) = \forall \overline{X'} \,.\, \mathit{1\text{-}pred}_{\mathcal{S}}(\overline{X'}) \rightarrow \mathit{unique\text{-}intersection}_{\mathcal{S}}(\overline{X},\overline{X'}). \tag{9}$$

Reasoning as before we obtain a refinement of Theorem 1 since every reachable marking has to satisfy both invariants.

**Theorem 2.** *Given a component-based system $\mathcal{S}$ and a* WS$\kappa$S *formula $\varphi(\overline{X})$, if the formula:*

$$\exists \overline{X} \,.\, \mathit{marking}_{\mathcal{S}}(\overline{X}) \wedge \mathit{1\text{-}invariant}_{\mathcal{S}}(\overline{X}) \wedge \mathit{trap\text{-}invariant}_{\mathcal{S}}(\overline{X}) \wedge \neg\varphi(\overline{X}) \tag{10}$$

*is unsatisfiable, then for every universe $\mathfrak{U}$, the property defined by the formula $\varphi(\overline{X})$ holds in every reachable marking of $\mathcal{N}_{\mathcal{S}}^{\mathfrak{U}}$.*

### 5 Experiments

We have implemented a prototype (called `ostrich` [15]) of this verification procedure to evaluate the viability of our approach. The current version of the prototype can only handle token-ring and pipeline topologies, but not trees; for these topologies the verification reduces to checking satisfiability of a formula of WS1S. We have also considered one example with tree-topology (see below), for which the formula was constructed manually. Satisfiability of WS1S and WS$\kappa$S formulae was checked using version 1.4/17 of Mona [33]. We consider various examples separated in categories:

**Cache Coherence.** Following [24] we formalized and checked the described safety properties and deadlock-freedom of the following cache coherence protocols: Illinois, Berkeley, Synapse, Firefly, MESI, MOESI, and Dragon.

**Mutual Exclusion.** We modelled and checked for deadlock-freedom and mutual exclusion Burns' [35], Dijkstra's and Szymanski's [3] algorithms as well as a formulation of Dijkstra's algorithm on a ring structure with token passing [30]. Furthermore, we check synchronization via a semaphore which is atomically aquired and by broadcasting to ensure everyone else is not in the critical section.

**Dining Philosophers.** This is the classical problem of dining philosophers which all take first the right fork and then the left fork. We consider the following "flavors" of this problem:

- there is one philosopher who takes first her left and then her right fork,
- as above but the forks remember whom took them, and
- there are two global forks everyone grabs in the same order.

**Preemptive Tasks.** There are tasks which can be either waiting, ready, executing or preempted. Initially one task is executing while all others are waiting. At any point a task may become ready and any ready task may preempt the currently executing task. Upon finishing the executing task re-enables one preempted task. Here, we have additionally two alternatives: Firstly, we consider the case where always the agent with highest index resumes execution. Secondly, we let the processes establish the initial condition from a position where everyone is waiting (referenced later as *uninitialized*).

**Dijkstra-Scholten.** This is an algorithm that is used to detect termination of distributed systems by message passing along a tree [25]. Since the prototype only supports linear topologies we can generate the necessary formula automatically only for this case.

**Herman.** This algorithm implements self-stabilizing token passing in rings. The formulation is modelled after [19]. This applies for all following examples. Hence, we describe the examples only in little detail.

**Israeli-Jalfon.** This is another self-stabilizing token passing algorithm in rings.

**Lehmann-Rabin.** This is a randomized solution to the dining philosophers problem.

**Dining Cryptographers.** A group of cryptographers want to determine if one of them paid for a meal or a stranger but do not reveal how they acted individually.

The results are shown in Table 1. The first column reports the size of the example in terms of the amount of states (#st.) and clauses (#cls.). The second column indicates which properties could (✓) and could not be verified (×) because the conjunction of trap and one-invariant was not strong enough to prove the given property. The third column reports the time (in second) it takes to prove all considered properties. These results are measured on the provided virtual machine for artifacts [32] where the host system is an average laptop. To understand the next four columns, recall that Mona constructs for a given formula $\phi(X)$ a finite automaton recognizing all the sets $X$ for which $\varphi$ holds. Since the automaton can have very large alphabets, its transition relation is encoded as a binary decision diagram (BDD). The columns report the number of states and the number of nodes of the BDD for different formulas. More precisely, the columns trap, trap-inv, flow, and flow-inv give the sizes of the automata for the formula $\mathit{trap\text{-}pred}_{\mathcal{S}} \wedge \mathit{initially\text{-}marked}_{\mathcal{S}}$, $\mathit{trap\text{-}invariant}_{\mathcal{S}}$, $\mathit{1\text{-}pred}_{\mathcal{S}}$ and $\mathit{1\text{-}invariant}_{\mathcal{S}}$ respectively. We write "n.a." (for "not available") to indicate that Mona timed out before the automaton was computed.

The first observation is that the satisfiability checks often can be done in very short time. This is surprising, because the formulas to be checked, namely (7) and (10), exhibit one quantifier alternation (recall that $\mathit{trap\text{-}invariant}_{\mathcal{S}}$ and $\mathit{1\text{-}invariant}_{\mathcal{S}}$ contain universal quantifiers). More specifically, since $\mathit{trap\text{-}invariant}_{\mathcal{S}}$ is obtained by universally quantifying over $\mathit{trap\text{-}pred}_{\mathcal{S}} \wedge \mathit{initially\text{-}marked}_{\mathcal{S}}$, one would expect the automaton for the former to be much larger than the one for the latter, at least in some cases. But this does not happen: In fact, the automaton for $\mathit{trap\text{-}invariant}_{\mathcal{S}}$ is almost always smaller. Similarly, there is no blowup from $\mathit{1\text{-}pred}_{\mathcal{S}}$ to $\mathit{1\text{-}invariant}_{\mathcal{S}}$. A possible explanation could be that the exponential blowup caused by universal quantification in $\mathsf{WS}\kappa\mathsf{S}$ manifests only on theoretical corner cases, which do not occur in our examples.

### 6 Conclusions

We have shown that the trap technique used in [11,28,13] for the verification of single systems can be extended to parameterized systems with sophisticated communication structures, like pipelines, token rings and trees. Our extension constructs a parameterized trap invariant, a formula of $\mathsf{WS}\kappa\mathsf{S}$ satisfied by the reachable global states of all instances of the system. The core of the approach is a purely syntactic, automatic derivation of the trap invariant from the interaction formula describing the possible transitions of the system. When the set of safe global states can also be expressed in $\mathsf{WS}\kappa\mathsf{S}$, which is usually the case, we check using the Mona tool whether the trap invariant implies the safety formula. The technique proves correctness of systems that do not produce well-structured transition systems in the sense of [1,29], and of systems with broadcast communication, for which, to the best of our knowledge, cut-off results have not been obtained yet.

Our experiments demonstrate that trap invariants can be very effective in finding proofs of correctness (inductive invariants) of common benchmark examples. In practice, the technique is very cheap, since it avoids costly fixpoint computations. This suggests incorporating it into other verifiers as a preprocessing step.

**Data Availability Statement and Acknowledgements.** The work of the second and fifth author has received funding from the European Research Council (ERC) under the European Union's Horizon 2020 research and innovation programme under grant agreement No 787367 (PaVeS).
The tool `ostrich` and associated files are available in the Zenodo repository: https://zenodo.org/record/3676940

| Benchmark | size #st. / #cls. | Properties | | time (s.) | trap #st. / #tr. | trap-inv #st. / #tr. | flow #st. / #tr. | flow-inv #st. / #tr. |
|---|---|---|---|---|---|---|---|---|
| Berkeley | 4 / 8 | deadlock-freedom<br>consistency properties | ✓<br>✓ | 0.37 | 18 / 95 | 18 / 78 | 12 / 50 | 7 / 18 |
| Dragon | 5 / 24 | deadlock-freedom<br>consistency properties | ✓<br>✓ | 1.63 | 39 / 433 | 32 / 159 | 54 / 537 | 11 / 57 |
| Firefly | 5 / 13 | deadlock-freedom<br>consistency properties | ✓<br>✓ | 0.53 | 55 / 409 | 36 / 200 | 38 / 309 | 11 / 44 |
| Illinois | 4 / 13 | deadlock-freedom<br>consistency properties | ✓<br>✓ | 0.46 | 14 / 83 | 11 / 32 | 16 / 95 | 9 / 38 |
| MESI | 4 / 8 | deadlock-freedom<br>consistency properties | ✓<br>✓ | 0.36 | 12 / 62 | 11 / 32 | 12 / 49 | 7 / 18 |
| MOESI | 5 / 10 | deadlock-freedom<br>consistency properties | ✓<br>✓ | 0.56 | 20 / 150 | 16 / 64 | 12 / 57 | 7 / 20 |
| Synapse | 3 / 6 | deadlock-freedom<br>consistency properties | ✓<br>✓ | 0.32 | 12 / 44 | 11 / 30 | 12 / 42 | 7 / 16 |
| Dijkstra-Scholten | 4 / 6 | deadlock-freedom | ✓ | 0.25 | 13 / 48 | 11 / 31 | 10 / 35 | 9 / 31 |
| Bakery | 3 / 4 | deadlock-freedom<br>mutual exclusion | ✓<br>✓ | 0.26 | 10 / 27 | 10 / 24 | 8 / 23 | 7 / 20 |
| Burns | 6 / 12 | deadlock-freedom<br>mutual exclusion | ✓<br>✓ | 0.30 | 10 / 64 | 9 / 30 | 8 / 35 | 7 / 32 |
| Dijkstra | 12 / 14 | deadlock-freedom<br>mutual exclusion | ✓<br>✓ | 11.86 | 375 / 11840 | 106 / 2887 | 13 / 148 | 10 / 110 |
| Broadcast MutEx | 2 / 2 | deadlock-freedom<br>mutual exclusion | ✓<br>✓ | 0.23 | 9 / 22 | 9 / 21 | 8 / 19 | 7 / 16 |
| Preemptive (high) | 5 / 4 | deadlock-freedom<br>mutual exclusion | ✓<br>✓ | 0.26 | 41 / 279 | 17 / 71 | 16 / 88 | 10 / 46 |
| Preemptive | 5 / 4 | deadlock-freedom<br>mutual exclusion | ✓<br>✓ | 0.25 | 28 / 173 | 22 / 97 | 20 / 113 | 12 / 62 |
| Preemptive (uninitialized) | 3 / 3 | deadlock-freedom<br>mutual exclusion | ✓<br>× | 0.25 | 20 / 80 | 11 / 37 | 8 / 23 | 7 / 20 |
| Semaphore | 4 / 2 | deadlock-freedom<br>mutual exclusion | ✓<br>✓ | 0.23 | 14 / 39 | 9 / 32 | 10 / 36 | 8 / 30 |
| Szymanski | 15 / 31 | deadlock-freedom<br>mutual exclusion | n.a.<br>n.a. | 19.35 | 495 / 34990 | n.a. | 8 / 84 | 7 / 66 |
| Dijkstra (ring) | 10 / 9 | deadlock-freedom<br>mutual exclusion | ✓<br>✓ | 75.63 | 703 / 10160 | 1149 / 23195 | 20 / 247 | 20 / 387 |
| Dining Cryptographers | 7 / 15 | deadlock-freedom<br>correctness | ×<br>✓ | 1.54 | 250 / 3232 | 288 / 2484 | 10 / 72 | 9 / 60 |
| Dining Philosophers (global) | 5 / 3 | deadlock-freedom | ✓ | 0.23 | 32 / 145 | 19 / 98 | 15 / 77 | 11 / 56 |
| Herman (linear) | 3 / 3 | deadlock-freedom<br>no token loss | ×<br>✓ | 0.25 | 19 / 70 | 14 / 42 | 10 / 33 | 9 / 27 |
| Herman (ring) | 3 / 4 | deadlock-freedom<br>no token loss | ✓<br>✓ | 0.26 | 19 / 71 | 14 / 42 | 10 / 33 | 9 / 27 |
| Israeli-Jalfon | 3 / 5 | deadlock-freedom<br>no token loss | ✓<br>✓ | 0.25 | 43 / 187 | 14 / 42 | 8 / 23 | 7 / 20 |
| Dining Philosophers (lefty) | 5 / 5 | deadlock-freedom | ✓ | 0.25 | 37 / 219 | 27 / 167 | 12 / 63 | 11 / 57 |
| Dining Philosophers (lefty, rem. forks) | 6 / 5 | deadlock-freedom | ✓ | 0.26 | 37 / 270 | 21 / 119 | 14 / 101 | 11 / 66 |
| Lehmann-Rabin | 6 / 7 | deadlock-freedom | ✓ | 0.26 | 39 / 361 | 23 / 211 | 11 / 67 | 11 / 73 |

Table 1: Experimental results of `ostrich`.
